# Proposed mechanisms of tau: relationships to traumatic brain injury, Alzheimer’s disease, and epilepsy

**DOI:** 10.3389/fneur.2023.1287545

**Published:** 2024-01-05

**Authors:** Samantha P. Martin, Beth A. Leeman-Markowski

**Affiliations:** ^1^Comprehensive Epilepsy Center, New York University Langone Health, New York, NY, United States; ^2^Department of Neurology, New York University Langone Health, New York, NY, United States; ^3^New York University Grossman School of Medicine, New York, NY, United States; ^4^VA New York Harbor Healthcare System, New York, NY, United States

**Keywords:** epilepsy, Alzheimer’s disease, tau phosphorylation, amyloid-beta, TBI, endoplasmic reticulum stress

## Abstract

Traumatic brain injury (TBI), Alzheimer’s disease (AD), and epilepsy share proposed mechanisms of injury, including neuronal excitotoxicity, cascade signaling, and activation of protein biomarkers such as tau. Although tau is typically present intracellularly, in tauopathies, phosphorylated (p-) and hyper-phosphorylated (hp-) tau are released extracellularly, the latter leading to decreased neuronal stability and neurofibrillary tangles (NFTs). Tau cleavage at particular sites increases susceptibility to hyper-phosphorylation, NFT formation, and eventual cell death. The relationship between tau and inflammation, however, is unknown. In this review, we present evidence for an imbalanced endoplasmic reticulum (ER) stress response and inflammatory signaling pathways resulting in atypical p-tau, hp-tau and NFT formation. Further, we propose tau as a biomarker for neuronal injury severity in TBI, AD, and epilepsy. We present a hypothesis of tau phosphorylation as an initial acute neuroprotective response to seizures/TBI. However, if the underlying seizure pathology or TBI recurrence is not effectively treated, and the pathway becomes chronically activated, we propose a “tipping point” hypothesis that identifies a transition of tau phosphorylation from neuroprotective to injurious. We outline the role of amyloid beta (Aβ) as a “last ditch effort” to revert the cell to programmed death signaling, that, when fails, transitions the mechanism from injurious to neurodegenerative. Lastly, we discuss targets along these pathways for therapeutic intervention in AD, TBI, and epilepsy.

## 1 Introduction

TBI and CTE are characterized by abnormal tau deposition in brain tissue. Epilepsy can also represent a form of tauopathy, as a result of cellular injury due to repetitive seizures. Seizure-induced injury responses include neuronal excitotoxicity and inflammatory cascades, which can lead to tau deposition and cell death (1–3). Tau is crucial for neuronal structural integrity and intracellular axonal transport (4, 5). Although tau is most commonly present intracellularly, p-tau is also found in the synaptic cleft (6, 7). Hp-tau leads to decreased neuronal stability and extracellular NFT formation, seen in neurodegenerative disorders including AD, CTE, TBI, and epilepsy. Tau cleaved by caspases, a family of enzymes involved in programmed cell death, is also present in NFTs (8, 9). Tau cleavage at specific sites by caspases increases susceptibility to hyper-phosphorylation and NFT formation, suggesting that cell death pathways contribute to the pathology of tauopathies (9).

The role of inflammation in this cascade, however, is unknown. We briefly outline key inflammatory proteins involved in molecular signaling in TBI, AD, and epilepsy; discuss ER stress and its differing roles in TBI, AD, and epilepsy; and summarize how inflammatory signaling imbalances the ER stress response post-injury. We propose that, in response to acute moderate–severe TBI or single seizures, both inflammatory signaling and an overwhelmed ER stress response activate tau-induced signaling pathways to prevent further cellular dysfunction and restore intracellular homeostasis. Furthermore, we propose that in response to repeated injury, there is chronic activation of pro-inflammatory pathways and continual imbalance of the ER stress response, along with chronic activation of tau-induced signaling pathways.

We discuss three distinct processes, neuroprotection, injury, and degeneration, where injury is potentially reversible, and degeneration represents the spread of toxic effects to neighboring neurons and a lower likelihood of reversibility. We propose pathways by which the neuroinflammatory response to injury (seizures or TBI) contributes to tau hyper-phosphorylation and NFT formation, ultimately presenting our final hypothesis: tau phosphorylation plays a key role in neuroprotection, responding to recurrent seizures/injury, but there is a “tipping point” from neuroprotective to injurious effects – the repeated or sustained induction of an imbalanced ER stress response (specifically, the unfolded protein response [UPR]) and tau phosphorylation/hyper-phosphorylation. The ER stress response stimulates tau phosphorylation and continued tau cleavage; further phosphorylation/hyper-phosphorylation of tau promotes a continued UPR response and promotes neurodegeneration. This chronic dysregulation results in a shift from a tau-induced signaling pathway as a compensatory, neuroprotective response – which once reduced cellular dysfunction and attempted to restore apoptotic-necrotic dynamics and cellular homeostasis – to an injurious mechanism that is unable to maintain intracellular homeostasis, nor dynamically revert to mechanisms of programmed cell death (apoptosis).

Lastly, we propose a role for Aβ and outline its “last ditch effort” to mediate the injurious effects of excitotoxicity and chronic tau pathway activation, reverting the cell to pro-death signaling. However, due to (1) sustained UPR signaling interacting with tau and Aβ (2) the inability of reactive astrocytes and microglia to successfully break down toxic tau and Aβ aggregates, this leads to further tau hyper-phosphorylation resulting in NFT formation, as well as Aβ plaque accumulation – the hallmarks of neurodegeneration seen in AD pathology.

## 2 Injury response: molecular signaling

Inflammatory signaling, excitotoxic propagation, and ER stress play key roles in the atypical activation of cell death cascades and excessive phosphorylation of tau, resulting in downstream toxic tau aggregates and eventual neurodegeneration.

### 2.1 Inflammatory proteins and neurotransmission

Inflammatory proteins, including receptor-interacting kinases (e.g., RIP1/RIP3) and cytokines (e.g., interleukin-1 [IL-1], caspases), modulate inflammatory function and regulate forms of cell death such as necroptosis and apoptosis (10–12). Effects of inflammatory mediators are complex, in that they differ based on injury type, location, and chronicity. Even a single, acute TBI can cause sustained inflammatory signaling, measured by interleukin (IL) protein levels (13). A continued inflammatory response may lead to secondary neuronal injury and a decreased likelihood of spontaneous recovery over time, with persistent neuropsychological deficits. Additional injuries may contribute to chronic functional deficits, due to shortened recovery time between injuries and long-term neurodegeneration.

Neurotransmitters can modulate inflammatory responses in brain injury by disrupting pro-inflammatory cytokines, microglial production, and calcium signaling (14). Glutamate and γ-aminobutyric acid (GABA) are the major excitatory and inhibitory neurotransmitters, respectively. Glutamate release into the synaptic cleft occurs via calcium influx and intracellular calcium-dependent signaling (15). Once glutamate acts upon post-synaptic neurons, astrocytes collect and convert it to glutamine which is transported back to pre-synaptic neurons (16). Neuronal excitotoxicity due to altered glutamate and GABA receptor expression and function is evident in models of TBI (17, 18).

N-methyl-D-aspartate (NMDA) and α-amino-3-hydroxy-5-methyl-4-isoxazolepropionic acid (AMPA) are glutamate receptors responsible for neuronal influx of calcium in post-synaptic neurons. Table 1 summarizes NMDA and AMPA functions during typical neuronal depolarization and action potential propagation. The net effect of selectively activating these receptors and regulating their post-synaptic densities is to potentiate a non-toxic glutamate response, which promotes synaptic plasticity, long-term potentiation, and learning and memory (17, 19, 20). However, if these receptors are unselectively trafficked to/from key synaptic regions in brain injury, the result is an acute disruption of these signaling processes. In mechanical models of injury, down-regulation of the AMPA GluR2 and NMDA N2A receptors, along with up-regulation of the NMDA N2B receptor, lead to atypical calcium influx resulting in acute excitotoxic cell death (21–23).

**Table 1:** Typical functionality (during selective activation) of glutamate and GABA receptors.

Receptor	Subtype	Typical functionality (during selective activation)
NMDA	NR1	Glycine-dependent receptor deactivation Localizes with NR2
NMDA	NR2A	Enhancement of excitatory synapses Localizes with NR1 Responds and initiates LTP
NMDA	NR2B	Ca^2+^ influx mediation Prolongs Ca^2+^ influx Responds and initiates LTD
AMPA	GluR1	Upregulated density in LTP Phosphorylates in LTP Permits Na^+^ and Ca^2+^ permeability
AMPA	GluR2	Restricts Ca^2+^ permeability
GABA	A-δ	Inhibition of potentiated response Responds to changes in GABA concentrations

LTP, long-term potentiation; Ca^2+^, calcium; LTD, long-term depression; Na^+^, sodium.

### 2.2 Molecular signaling in TBI

Although TBI primarily leads to neocortical cell death, hippocampal vulnerability is also apparent. In a controlled cortical impact (CCI) mouse model of moderate TBI, apoptosis of immature hippocampal neurons was observed 24–72 h after injury (24). Limited inflammatory markers may be observed up to 7 days post-CCI, and necrosis of immature hippocampal neurons was evident for at least 14 days post-injury (25, 26). These results demonstrate hippocampal vulnerability in response to TBI that may clinically present as memory complaints.

Both altered excitatory glutamate signaling and reduced GABA-mediated inhibition contribute to excitotoxicity in brain injury (27). In a mouse CCI model, glutamate expression correlated with epileptiform activity within injured and adjacent cortex in the setting of decreased GABAergic interneurons. Further, there was significant reduction of the GABA_A_ γ2-subunit in CCI-injured rats with post-traumatic epilepsy (18). In a mouse CCI model of severe TBI, GABA_A_ δ and GABA_B_ B2 receptor subunit expression in dentate gyrus granule cells was reduced by 40–43% (24). In contrast, human studies of chronic, repetitive injuries in athletes (closed head injury [CHI] model) found a compensatory increase in GABA_B_ receptor expression (28). Decreased GABA_A_ receptor expression disrupts the inhibitory response (29), while increased GABA_B_ receptor expression, responsible for membrane hyperpolarization, may serve to avoid further depolarization and excitotoxic effects.

### 2.3 Molecular signaling in AD

AD pathology includes Aβ plaque accumulation and NFT formation, with tau aggregation and hyper-phosphorylation contributing to dysregulated microtubule dynamics and neuronal functioning (30). Necroptosis activation by RIP1/RIP3 kinases was found in postmortem AD brains (31). Elevated levels of inflammatory markers IL-1β, IL-6, and tumor necrosis factor-alpha (TNF-α) were found in postmortem AD and transgenic animal brains, and microglial and astrocytic activation was observed in response to neurotoxic cytokine expression (32–36).

Excitotoxicity due to dysregulated Ca^2+^-mediated NMDA receptor functioning decreases cell survival (37, 38). Aβ regulates synaptic vesicle release and affects NMDA receptor structure, density, and electrophysiology – ultimately affecting glutamate transmission and resulting in cognitive changes (39–43). In AD patients with severe cognitive deterioration, decreased glutamate and GABA levels were noted in temporal cortex and CSF compared to AD patients with mild cognitive deterioration and age-matched controls (44, 45), and decreased concentrations of GABAergic terminals in cortical neurons adjacent to Aβ plaques were found in AD patients and transgenic AD mouse models (46, 47). These findings suggest impaired receptor function and neurotransmission and an imbalance between excitatory and inhibitory activity in AD.

### 2.4 Molecular signaling in epilepsy

Inflammatory responses in epilepsy can contribute to recurrent seizures, secondary neuronal injury, and chronic neurodegeneration (2). During focal to bilateral tonic–clonic seizures, cytokines exert effects through increased AMPA receptor density, NMDA-dependent calcium influx, and reduction of GABA_A_ receptor density, resulting in greater synaptic glutamate and decreased synaptic GABA concentrations (48–51). Excess glutamate increases the likelihood of neuronal depolarization, excitotoxicity, and eventual cell death (52–54), particularly in models of temporal lobe epilepsy (55). Glia rapidly produce interleukins, particularly interleukin-1 beta (IL-1β), postictally. IL-1β enhances neuronal excitability and sustains inflammatory responses (56, 57). Increased IL-1β activity leads to neuronal degeneration in epileptogenic regions, while astrocytes that express its receptor have neuroprotective functions (1, 58). Astrocytes can mediate the effect of IL-1β on hippocampal neurons, contributing to their likelihood of survival. The presence of astrocytes in epileptogenic regions is a compensatory response to excess synaptic glutamate (59, 60). Increased astrocytes in regions of post-ictal neuronal injury suggest IL-1β involvement in the initiation and continuation of local seizure activity (59).

**Figure 1:**
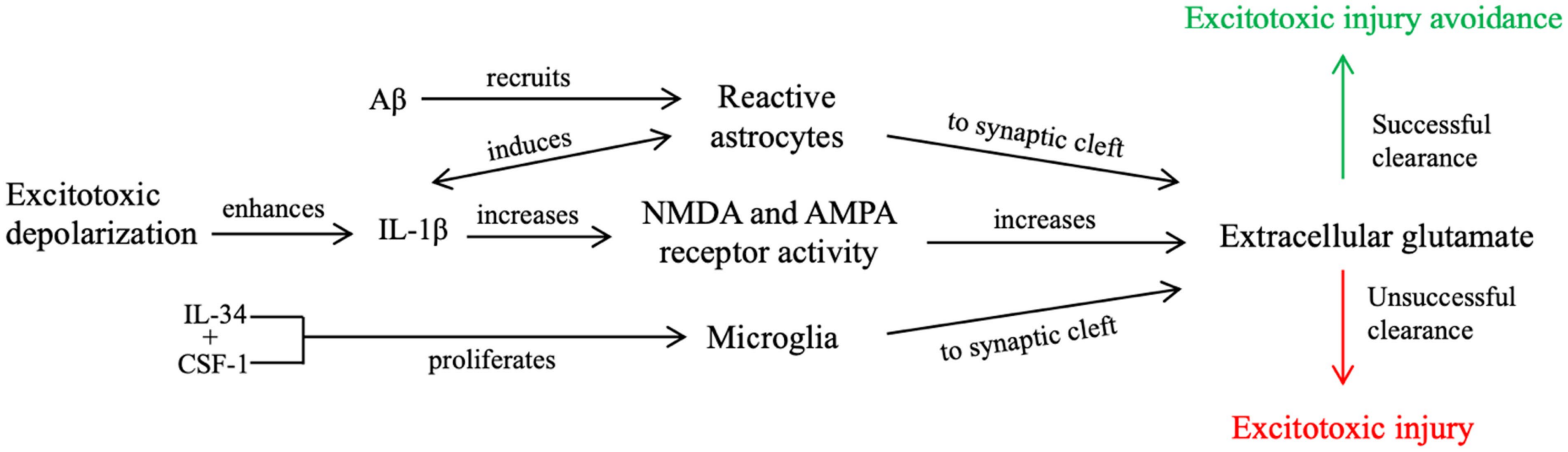
Our proposed contributory mechanism of IL-1β signaling during a single, brief seizure or mild TBI. Enhanced IL-1β signaling from excitotoxic depolarization results in increased glutamate receptor activity and further propagation of excitotoxic signaling, resulting in an accumulation of post-synaptic glutamate. Increased neuroinflammatory signaling, including upregulated cytokine and Aβ secretion and increased concentrations of extracellular glutamate, recruit microglia and reactive astrocytes to the post-synaptic cleft. Unsuccessful clearance of extracellular glutamate, cellular debris, and Aβ from the synaptic cleft by reactive astrocytes and microglia leads to further excitotoxic propagation and places the cell at risk for excitotoxic injury. Successful clearance, however, reduces the risk of excitotoxic injury, as it attempts to revert the cell to neuronal homeostasis.

We propose that during a single seizure and mild TBI (Figure 1), excitotoxic depolarization enhances IL-1β signaling and increases NMDA receptor activity, leading to local propagation of excitotoxic depolarization and extracellular glutamate accumulation. This process, along with increased Aβ and cytokine secretion, recruits astrocytes into the synapse (61) to collect glutamate post-seizure. Excess glutamate also recruits microglia to clear cellular debris, remove excess Aβ, and return to neuronal homeostasis (60, 62, 63). If neuronal homeostasis is not achieved, further excitotoxic injury and cell death signaling can occur.

Neuronal damage in TBI, AD, and epilepsy can result from secondary inflammatory responses and neuronal excitotoxicity. Interleukins, particularly IL-1β, are key modulators of pro-inflammatory responses and apoptosis. Additionally, dysregulation of the glutamate-GABA/excitation-inhibition balance leads to excitotoxic injury and neuronal death.

### 2.5 ER stress and its role in TBI, AD, and epilepsy

ER stress occurs when there is an imbalance between the ability of the ER to fold proteins and the cellular demand for protein folding (64). In response to ER stress, the UPR signals to either (1) protect the cell by correcting the imbalance between folding ability and demand (65) via the protein kinase R-like ER kinase (PERK) pathway or (2) promote programmed cell death. Cell death occurs via C/EBP homologous protein (CHOP) and Apaf-1-dependent apoptosis or via necroptosis involving RIP1/RIP3-activation and rapid ATP depletion (66–68). Acute UPRs are protective to the cell. Sustained UPRs, however, induce caspase-dependent apoptosis (69), deplete intracellular ATP (70), and induce necrosis (70).

ER stress contributes to neuronal loss in TBI (26, 71, 72), AD (73), and epilepsy (74) and correlates with tau phosphorylation in TBI and AD (75, 76). In a CCI rat model, markers of reactive ER stress were associated with increased tau oligomers and tau kinase (GSK-3β) activation (77). To study the relationship between tau phosphorylation and ER-stress in promoting AD-like pathogenesis, tau phosphorylation was induced in rat cortical neurons, resulting in a UPR response with elevation of p-PERK and other modulator proteins. In the same study, an ER stress inducer enhanced tau phosphorylation at specific sites (75).

In human AD autopsy material, PERK correlated with atypical tau phosphorylation (78), and tau interacted with ER proteins leading to neuronal dysfunction and neurotoxicity (79). In epilepsy, the relationship between ER stress and tau phosphorylation is unknown, although relationships between epilepsy and unfolded proteins have been established. A mouse model of epilepsy suggested that acute, reactive ER stress responses may reduce seizure recurrence or severity (80). In resected tissue from patients with epilepsy due to focal cortical dysplasia, however, there were greater accumulations of unfolded proteins and increased levels of CHOP in patients who were not rendered seizure-free (81). Hence, acute, reactive stress responses may be protective, while chronically increased ER stress may contribute to seizure recurrence.

Aβ can trigger ER stress, just as ER stress can promote Aβ formation, leading to excitotoxicity and apoptosis (82–84). While amyloid precursor protein (APP) increases resistance to ER stress-induced apoptosis in specific cell cultures (85), intracellular Aβ counteracts APP by activating ER stress and pre-disposing cells to other pathways of programmed cell death (86). In brain endothelial cells, Aβ increased concentrations of UPR signaling mediators, increased intracellular Ca^2+^, and upregulated pro-apoptotic transcription factors (87). The relationship between Aβ and excitotoxicity is complex, however, in that Aβ also acts directly on the ER stress response protein XBP1 to reduce intracellular Ca^2+^ concentrations and limit excitotoxic injury (88).

Data suggest initial neuroprotective effects of reactive ER stress, activation of the PERK pathway, and APP (89). However, we postulate that sustained, repeated, or anticipatory (i.e., in the face of chronic injury) induction of the ER stress response may increase atypical tau phosphorylation and Aβ concentrations, with deleterious effects. Aβ has both pro-apoptotic and excitotoxic effects, but to limit neural injury, it acts feeds back on the ER stress response to interrupt it. If Aβ fails to halt its excitotoxic effects, and microglia and reactive astrocytes cannot successfully clear toxic tau and Aβ aggregates, neurodegeneration follows.

## 3 Injury response: the role of tau

Tau plays a key role in ER stress and Aβ pathways. Tau is a neuronal protein that supports axonal transport and microtubule dynamics (4). In neurodegenerative diseases, tau is abnormally present within subcortical neurons, including the hippocampus. Tau hyper-phosphorylation results in deposits of neurofibrillary tangles (NFTs), corresponding with diminished neuronal stability and subsequent aberrant neuronal communication (4, 5). These structural abnormalities lead to cognitive deficits, including memory loss (90–93). Elevated levels of total- (t-), phosphorylated- (p-), and hyperphosphorylated- (hp-) tau are detected in CSF at various time points post-TBI/seizure (91, 94–98). Accumulation and spread of tau aggregates occurs in various cortical and subcortical areas post-injury/seizure and in AD (93, 94, 99–101).

**Figure 2:**
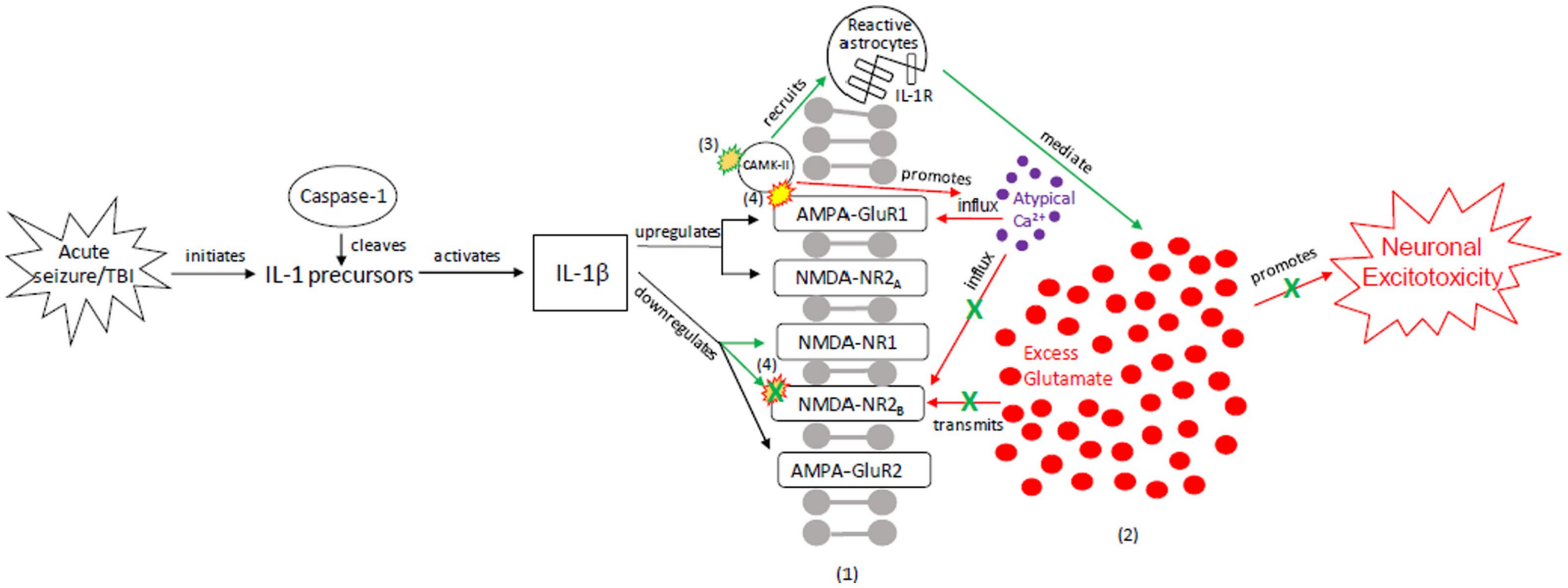
Our proposed acute injury response (AIR) mechanism outlining reactive signaling to an acute, brief seizure or acute, mild TBI. This mechanism shunts cellular signaling away from pro-death response pathways and toward cellular protection, with the goals of restoring the balance between glutamate release and reuptake, intracellular Ca^2+^-driven ER stress responses, and apoptotic-necrotic dynamics. In response to acute injury, caspase-1 cleaves IL-1 precursors, resulting in IL-1β formation. IL-1β unselectively up-or down-regulates glutamate receptor subunit densities, resulting in an acute disruption of balanced glutamate release and reuptake. There is increased CaMK-II activation that promotes increased glutamate release (102, 103). Unselective CaMK-II phosphorylation and autophosphorylation occurs at upregulated AMPA-GluR1 (104) but not at down-regulated NMDA-R2B (105), resulting in increased AMPA-GluR1 Ca2+ influx/channel conductance and decreased NMDA-NR2B Ca2+ influx/channel conductance, respectively. However, CaMK-II also recruits astrocytes into the affected region (59, 60, 106, 107). Increased astrocytes/IL-1 receptor density aid in clearing excess glutamate and ILs, inhibiting further glutamate release, thereby limiting excitotoxic propagation. (1) = Neuronal membrane, (2) = Synaptic cleft, (3) = CaMK-II autophosphorylation, (4) = CaMK-II-Glutamate receptor phosphorylation. Red = Excitotoxic signaling, Green = Neuroprotective signaling. X = response reduction/down-regulation.

**Figure 3:**
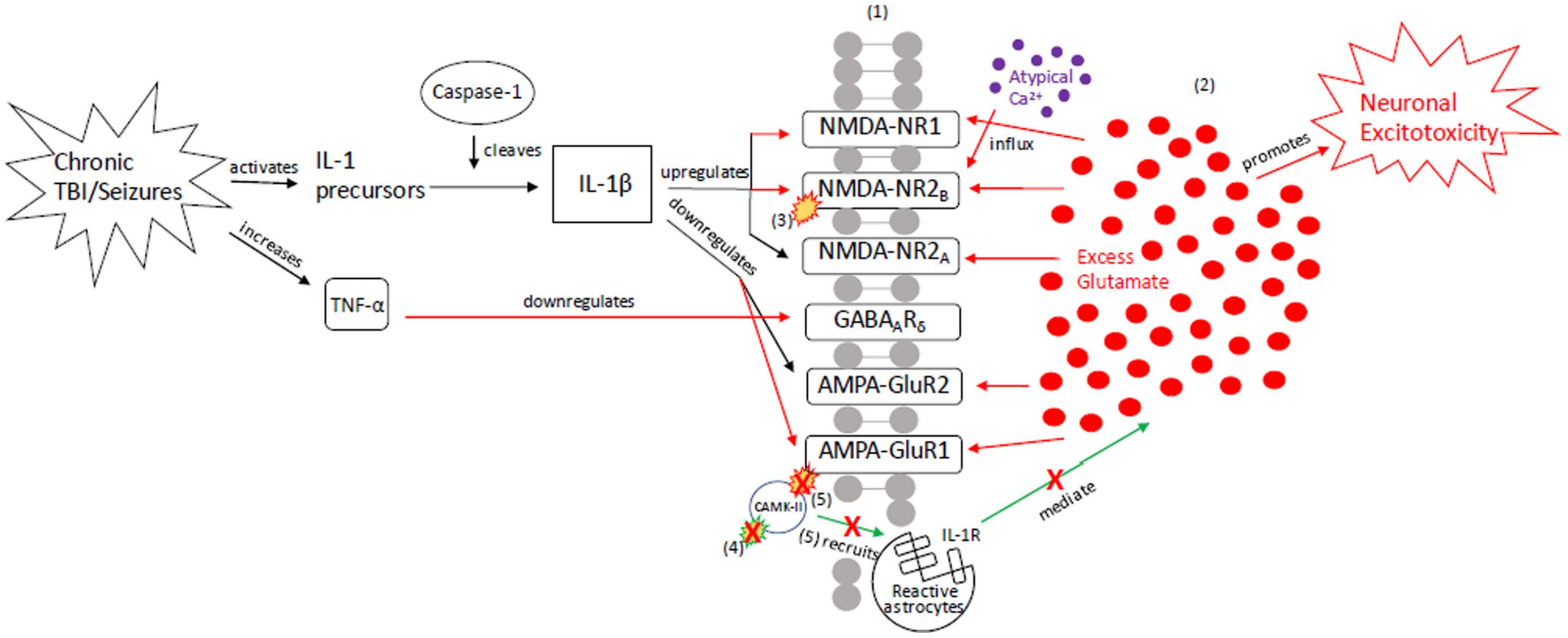
Our proposed recurrent injury response (RIR) mechanism outlining reactive signaling to chronic and/or moderate–severe TBI and chronic and/or prolonged seizures. This mechanism shunts cellular signaling toward pro-death response pathways of apoptosis and necrosis due to imbalanced glutamate release and reuptake, Ca^2+^-driven ER stress responses, and apoptotic-necrotic dynamics. In response to chronic injury, caspase-1 cleaves IL-1 precursors, resulting in IL-1β formation, and TNF-α downregulates GABA_A_ receptors (18, 108–113). However, unlike the AIR mechanism, IL-1β increases NMDA receptor activity via GluNR2B phosphorylation (112). Increased NMDA receptor densities contribute to atypical Ca^2+^ influx and prolonged excitotoxic signaling. Concurrently, AMPA-GluR1 and-GluR2 receptors are down-regulated in response to chronic injury, resulting in dysregulated CaMK-II autophosphorylation and AMPA-GluR1 site phosphorylation (21–23, 49, 114–116). Due to disrupted CaMK-II phosphorylation and autophosphorylation, reactive astrocytes cannot be successfully recruited to the synapse to clear excess glutamate and proteasome recruitment into dendritic spines is impaired, respectively (117). The result is neuronal excitotoxic depolarization and propagation, neurotoxic release of ATP, and preferential apoptotic signaling (118). (1) = Neuronal membrane, (2) = Synaptic cleft, (3) = IL-1β-activated NMDA-NR2B phosphorylation, (4) CaMK-II autophosphorylation, (5) CaMK-II-AMPA-GluR1 phosphorylation. Red = Excitotoxic signaling, Green = Neuroprotective signaling. X = response reduction/down-regulation.

**Figure 4:**
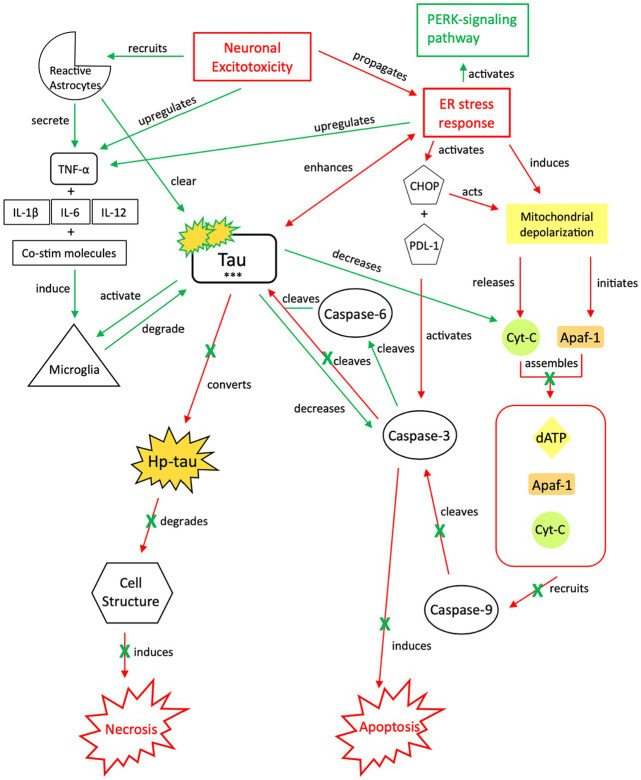
Our proposed neuroprotective response mechanism involving tau (NPT). Neuronal excitotoxicity imbalances the ER stress response, which activates two pathways: the PERK pathway, responsible for reverting the cell to homeostasis and preserving its integrity, and pro-cell death signaling cascades via CHOP and rapid mitochondrial depolarization, such as apoptosis. In typical apoptotic signaling, mitochondrial depolarization initiates Apaf-1 and releases cyt-c. Cyt-c, with Apaf-1 and dATP, assembles into an apoptosome complex (119–121). The apoptosome complex recruits caspase-9, caspase-9 cleaves caspase-3, and caspase-3 activates apoptosis (122, 123). Tau preserves cellular integrity and reverts cellular signaling away from pro-cell death signaling cascades. Although reduction of caspase-3 cleavage of tau reverts the cell away from apoptotic signaling, tau is cleaved by additional caspases such as caspase-6, resulting in tau phosphorylation (124–128). The increased presence of p-tau decreases the concentration of cyt-c and caspase-3, thereby further inhibiting apoptotic signaling (122, 129, 130). To avoid additional cell death pathways (i.e., necrosis), increases in cytokine expression, TNF-α, and tau concentrations recruit reactive astrocytes and microglia to break down excess tau into non-toxic components (131–136). Successful breakdown of accumulated tau by microglia and reactive astrocytes downregulates pro-death signaling pathways and restores cellular homeostasis. PERK, protein kinase R-like ER kinase; TNF, tumor necrosis factor; IL, interleukin; Co-stim, co-stimulatory (molecules); cyt-c, cytochrome-c; Apaf-1, apoptotic peptidase activating factor-1; dATP, deoxyadenosine triphosphate; NFTs, neurofibrillary tangles; Red, Pro-death signaling; Green, Neuroprotective signaling; o-tau, tau oligomers; t-tau, total tau; p-tau, phosphorylated tau; ***, O-tau, t-tau, p-tau; X, response reduction/down-regulation.

To explain the role of tau in brain injury and its relationship to the above inflammatory and excitotoxic processes, we posit two distinct signaling mechanisms, combining components of various pathways described in the literature: (1) an acute injury response (AIR; Figure 2), and (2) a recurrent injury response (RIR; Figure 3). AIR and RIR propose varying degrees of interleukin, NMDA/AMPA receptor, and Ca^2+^/calmodulin-dependent protein kinase (CaMK) involvement. We also propose a slower neuroprotective tau (NPT) response mechanism shared by acute seizures and TBI. However, with repeated seizures/TBI leading to chronically activated/sustained ER stress responses, the NPT pathway will become dysregulated, resulting in neural injury (Figure 4).

### 3.1 Acute injury response (AIR)

The AIR pathway is a pro-inflammatory mechanism that minimizes the likelihood of acute excitotoxic effects and cell death. In the AIR pathway, an acute TBI or brief seizure leads to IL-1β formation (108–110, 122), which has multiple effects on NMDA and AMPA receptors (Figure 2), including downregulation of NMDA receptors NR1 and NR2B. Unselective CAMK-II activation, coupled with the IL-1β signaling, promotes atypical calcium influx and excitotoxic glutamate release. As a result, there is an increased probability of cell death unless excess glutamate can be cleared from the synapse. CAMK, however, also recruits astrocytes into the affected region, evidenced by reactive astrocytes and phosphorylated CAMK-II in the hippocampal CA3 region of a kainic acid mouse model (106). The inflow of reactive astrocytes, coupled with increased IL-1 receptor density, clears excess synaptic glutamate (59, 60, 107).

### 3.2 Recurrent injury response (RIR)

The RIR pathway results in excitotoxity and apoptosis (Figure 3). Recurrent TBI activates -IL-1 precursors, which are cleaved into IL-1β by proteases such as caspase-1 (108–110). Similarly, recurrent seizures, through excitotoxic neuronal depolarization, activate caspase-1 and lead to IL-1β signaling (108–111). IL-1β, however, does not down-regulate NMDA receptors as in AIR. Instead, IL-1β hyper-activates NMDA receptors via GluNR2B subunit phosphorylation in response to chronic injury (112). The resultant increase in NMDA receptor density contributes to atypical calcium influx, prolongs excitatory synaptic enhancement, and propagates pathologic signaling from excess glutamate.

Further, there is decreased GABA_A_ receptor density (50) and downregulation of the GABA_A_ receptor δ-subunit (18, 113), contributing to extracellular glutamate accumulation and excitotoxicity (18, 113). AMPA-GluR1 and GluR2 receptors are also down-regulated in response to injury (21–23, 49, 114–116). As a result of AMPA dysregulation, CAMK-II autophosphorylation is impaired and recruitment of proteasomes – highly active enzyme complexes that play a role in cell-cycle progression – into dendritic spines is blocked, resulting in apoptosis (117). Additionally, subsequent phosphorylation at AMPA receptors also indirectly decreases astrocytic recruitment and clearance of excess glutamate (118).

If the AIR pathway (Figure 2) is unsuccessful in mediating excitotoxicity or if the RIR pathway is activated in chronic injury/seizures (Figure 3), apoptosis (acute programmed cell death) and necrosis (passive cellular degradation and death) result (142). Oxygen free radical production, caspase activation (e.g., caspase-3 and caspase-6), mitochondrial membrane depolarization, and further neurotoxicity occur (143–145). To minimize the possibility of cell death and preserve structural and functional integrity of surrounding neurons, an additional neuro-protective response is needed. We posit that tau signaling pathways first respond to recurrent seizures/injury in attempt to preserve cellular integrity; however, there is a “tipping point” that transitions the mechanism from neuroprotective to injurious – the repeated or sustained induction of an imbalanced ER stress response (specifically, the unfolded protein response [UPR]) and resultant aberrant tau phosphorylation. The ER stress response stimulates tau phosphorylation and continued tau cleavage; further phosphorylation/hyper-phosphorylation of tau promotes a continued UPR response and promotes neurodegeneration. This chronic dysregulation results in a shift from a tau-induced signaling pathway as a compensatory, neuroprotective response – which once reduced cellular dysfunction and restored apoptotic-necrotic dynamics and cellular homeostasis – to an injurious mechanism that is unable to maintain intracellular homeostasis, nor revert to mechanisms of programmed cell death.

### 3.3 Neuroprotective response (NPT): the expression and consumption of tau

In apoptosis, caspase-3 is activated by multiple mechanisms, including inflammatory responses, mitochondrial-based pathways, and an imbalanced ER stress response (119–123) (Figure 4). To divert the cell away from this apoptotic pathway and attempt to restore cellular homeostasis while maintaining structural integrity, caspases and ATP processes that induce apoptosis must be downregulated, TNF-α expression must be promoted, and tau phosphorylation must be induced, in conjunction with ER stress-induced PERK-pathway activation. Decreasing available caspases and apoptotic signaling reduces the likelihood of further neurotoxic depolarization and cell death, while increasing the likelihood that cellular homeostasis is restored (146). Induction of tau phosphorylation via caspase-6 cleavage indirectly reduces apoptotic signaling while preserving cellular integrity; tau also indirectly activates microglia, which are responsible for tau degradation to its non-toxic components.

Both caspase-3 and caspase-6 cleave tau (124–126) at multiple sites, which increases the susceptibility of tau to phosphorylation (9, 126–128). However, increased tau phosphorylation will also decrease caspase-3 activation in a negative feedback loop (122, 129, 130, 147). We posit that although the imbalanced ER stress response induces atypical tau phosphorylation (75), its acute effect is minimal due to this reduction in caspase-3 activation. As caspase-3 activation is required by apoptosis (119–121, 148), we posit that there is a transition from apoptosis to cellular preservation. However, with a halt of apoptotic signaling in the setting of increased tau concentrations, microglial and reactive astrocyte activation via upregulation of TNF-α, pro-inflammatory cytokines (IL-1β, IL-6, IL-12) and enzymes, and co-stimulatory molecules (131, 132) is required to break down tau. Additionally, tau oligomers (o-tau) and aggregates activate microglia to phagocytize tau and process its isoforms into non-toxic components (133–136). The ER stress response also upregulates Ca^2+^-ATPases in microglia, enhancing their capacity for phagocytosis and tau breakdown (149). Tau clearance is crucial to reestablishing cellular homeostasis and re-balancing the ER stress response post-seizure/injury.

### 3.4 Neuro-injurious tau response (NIT): transitioning from neuroprotection to injury

**Figure 5:**
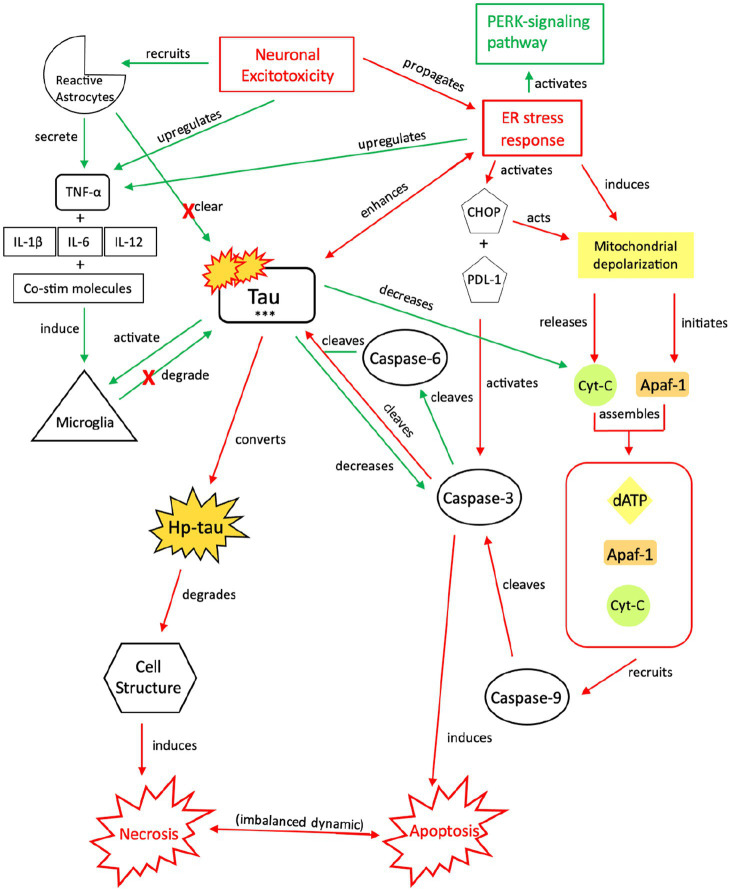
Our proposed injurious response mechanism involving tau (NIT) outlining injurious tau signaling and the resulting imbalance in apoptotic-necrotic signaling due to a chronic or sustained injury response from TBI or seizures. Similar to the NPT response, neuronal excitotoxicity imbalances the ER stress response, which activates two pathways: the PERK pathway and pro-cell death signaling pathway. Increased presence of p-tau decreases the concentration of cyt-c and caspase-3, inhibiting apoptotic signaling; although downregulated, caspase-3 still cleaves tau and contributes to tau’s toxic effects, which further reverts the cell away from apoptotic signaling toward necrosis (119–121). To compensate for this shift, increased cytokine expression, increased TNF-α, and increased tau concentrations recruit reactive astrocytes and microglia to break down excess tau into non-toxic components (107, 131, 132). However, unsuccessful breakdown of tau by microglia and reactive astrocytes results in a build-up of toxic tau aggregates that are secreted extracellularly (137, 138). Adjacent cells attempt to break down the toxic tau into non-toxic components (99), but chronic activation of the NIT pathway due to recurrent or sustained injury dysregulates this response, resulting in an injurious build-up of toxic levels of tau, hp-tau, and NFTs, which reinforce necrotic signaling (139–141). PERK, protein kinase R-like ER kinase; TNF, tumor necrosis factor; IL, interleukin; Co-stim, co-stimulatory (molecules); cyt-c, cytochrome-c; Apaf-1, apoptotic peptidase activating factor-1; dATP, deoxyadenosine triphosphate; NFTs, neurofibrillary tangles; Red, Pro-death signaling; Green, Neuroprotective signaling; o-tau, tau oligomers; t-tau, total tau; p-tau, phosphorylated tau; ***, O-tau, t-tau, p-tau; X, response reduction/down-regulation.

We posit that in an acute, mild TBI or brief seizure, tau will assist the cell in reverting to balanced ER stress response signaling and intracellular homeostasis. However, chronic or sustained activation of tau signaling cascades due to severe and/or recurrent injury will eventually transition this mechanism from neuroprotective to injurious (Figure 5). While tau expression benefits microtubule dynamics, overexpression of phosphorylated, cleaved isoforms disrupts microtubule transport and increases the risk of toxic tau aggregates (150). The overexpression of tau, atypical accumulation of p-tau and hp-tau from caspase-3 cleavage and apoptosis inhibition, and tau deposition due to the inability of microglia to successfully break down toxic tau aggregates, could be a result of the cell’s failed attempt to maintain homeostatic microtubule dynamics.

The NPT process depends upon the ability of the cell to revert to balanced ER stress responses, balanced apoptotic-necrotic dynamics, and intracellular homeostasis. Successful reactive astrocytic phagocytosis of tau and microglial clearance of tau play key roles in restoring intracellular dynamics. We posit that in the setting of sustained or recurrent injury, however, the ability of reactive astrocytes and microglia to break down tau becomes dysregulated. A resultant buildup of intra-microglial toxic tau occurs (99), which inhibits microglial and reactive astrocytic phagocytosis, threatens neuronal integrity, and drives expulsion of toxic tau aggregates from the cell via exosomal packaging and secretion. However, these secreted toxic tau aggregates are misfolded (151) and therefore more resistant to microglial break down. These exosomal tau aggregates have injurious effects (99) due to increased likelihood of exosomal leakage and surrounding neuronal uptake (152, 153). Further, the recurrent or sustained activation of the ER stress response reinforces microglial migration and dysregulation and limits the ability of microglia to actively break down tau. The inter-neuronal spread of toxic tau may mark the initial transition from a neuroprotective to a more widespread injurious process.

The NPT response may be an attempt to preserve cellular integrity, by avoiding further injury from apoptosis through tau phosphorylation and limiting effects of necrosis through astrocytic and microglial involvement. Over time, however, the NPT mechanism will still result in cell death if the underlying chronic pathology remains untreated. Further, with recurrent injury (e.g., repetitive seizures, repeated head trauma), the NPT response will become overwhelmed, and an aberrant, injurious process will ensue. Over time, repeated activation of injurious pathways will require a “last ditch effort” to revert the cell to pro-apoptotic signaling cascades and avoid further transition to a widespread neurodegenerative process, which leaves the question – what is the role of Aβ?

## 4 The role of amyloid-β in the transition from neuroprotection to tauopathy

**Figure 6:**
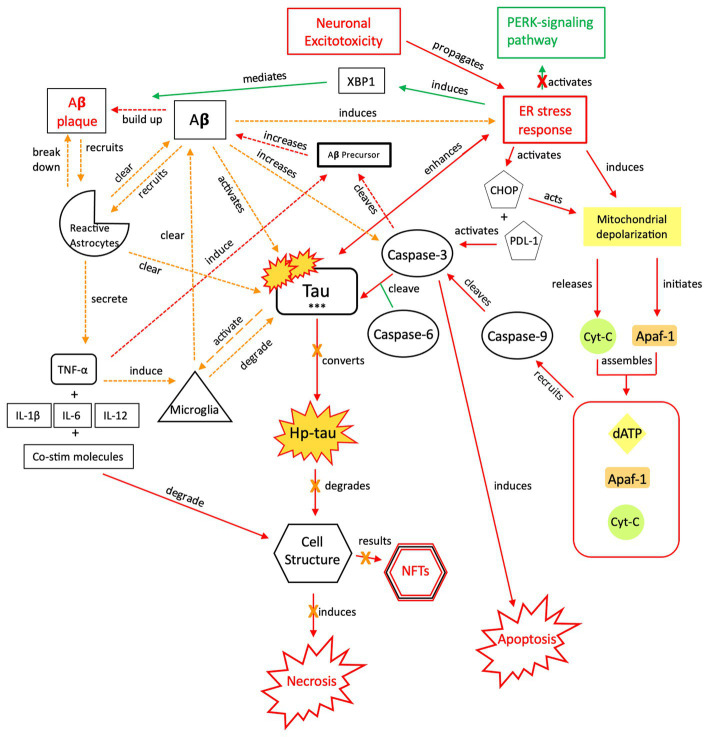
The neuroprotective response of Aβ, aka the “last ditch effort” to revert the cell to programmed death signaling and rebalance the apoptotic-necrotic signaling dynamic. In response to a recurrent or sustained ER stress response, imbalanced apoptotic-necrotic signaling dynamic, and atypical tau phosphorylation, Aβ activation both induces the ER stress response and increases caspase-3 cleavage of Aβ precursor protein (155). However, Aβ also recruits microglia and reactive astrocytes in response to excitotoxic signaling and increased tau concentrations (156). Breakdown of toxic tau aggregates and Aβ by microglia and reactive astrocytes mitigates the effect of Aβ-associated tau seeding and propagation (133, 157). As increased microglial trafficking is indirectly induced by the presence of Aβ, this mechanism also has detrimental effects due to shared apolipoprotein E (APOE), amyloidosis, and microglial transcript pathways and sustained neuroinflammation (158). Due to microglial inflammation and activation, reactive astrocytes are upregulated and recruited in attempts to clear toxic tau and Aβ and further orient the cell toward apoptotic signaling (159–162). Ultimately, a reduction in both inflammatory signaling and tau phosphorylation are needed once apoptotic-necrotic signaling dynamics have been reestablished, to prevent transition to an irreversible, degenerative pathway. PERK, protein kinase R-like ER kinase; Aβ, amyloid beta; XBP1, X-box binding protein 1; TNF, tumor necrosis factor; IL, interleukin; Co-stim, co-stimulatory (molecules); Cyt-c, cytochrome-c; Apaf-1, apoptotic peptidase activating factor-1; dATP, deoxyadenosine triphosphate; NFTs, neurofibrillary tangles; Red = Pro-death signaling, Green = Neuroprotective signaling, Orange = Aβ-involved signaling; o-tau, tau oligomers; t-tau, total tau; p-tau, phosphorylated tau; *** = O-tau, t-tau, p-tau. X = reduction/down-regulation. Solid line = signaling cascade induced/propagated by the ER stress response and tau; dashed line = signaling cascades resulting from Aβ involvement.

**Figure 7:**
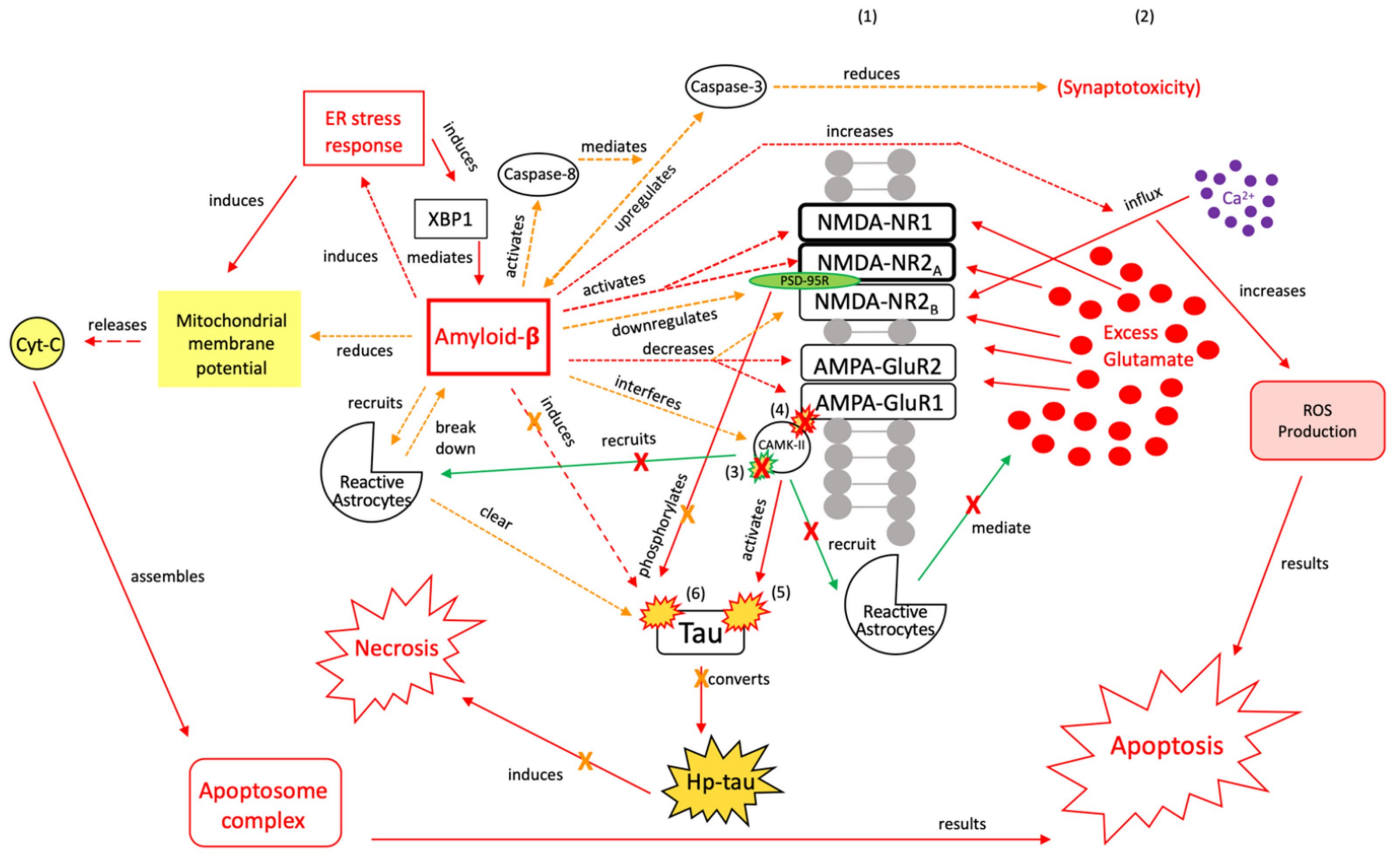
The neuroprotective response of Aβ, aka the “last ditch effort” to revert the cell to pro-apoptotic signaling and reduce tau and Aβ toxicity. Selective NMDA regulation, downregulating scaffolding protein PSD-95, and activating caspase-8 reduce the excitotoxic effects of Aβ and atypical tau phosphorylation (154). PSD-95 receptor downregulation results in reduced tau phosphorylation and protection of synapses from the effects of Aβ, while caspase-8 activation indirectly reduces synaptotoxicity by mediating the relationship between Aβ and caspase-3 (163, 164). Aβ also reduces mitochrondrial membrane potential and directly induces the ER stress response, resulting in apoptosome complex formation and eventual ROS-induced apoptosis (165). Reactive astrocytes are recruited to break down Aβ and clear tau aggregates. However, Aβ also has injurious effects, as it increases intracellular Ca^2+^ and ROS production, while also acting directly on tau (154). Therefore, this mechanism is considered a “last ditch effort” to acutely kill the cell via apoptotic signaling to minimize the negative effects from toxic tau, Aβ accumulation, and necrotic signaling. We posit that limiting the effects of tau and Aβ toxicity is predicated on the acute nature of this response and treatment of the underlying pathology to avoid irreversible injury and/or a transition to a more widespread neurodegenerative process. XBP1, X-box binding protein 1; Cyt-c, cytochrome-c; ROS, reactive oxygen species; Red = Pro-death signaling, Green = Neuroprotective signaling, Orange = Aβ-involved signaling; X = reduction/down-regulation. Solid line = signaling cascade induced/propagated by the ER stress response and tau; dashed line = signaling cascades resulting from Aβ involvement. (1) = Neuronal membrane, (2) = Synaptic cleft, (3) = CaMK-II-autophosphorylation, (4) = CaMK-II-Glutamate receptor phosphorylation, (5) = CaMK-II-tau-phosphorylation, (6) = PSD95-NMDA receptor complex-tau phosphorylation.

**Figure 8:**
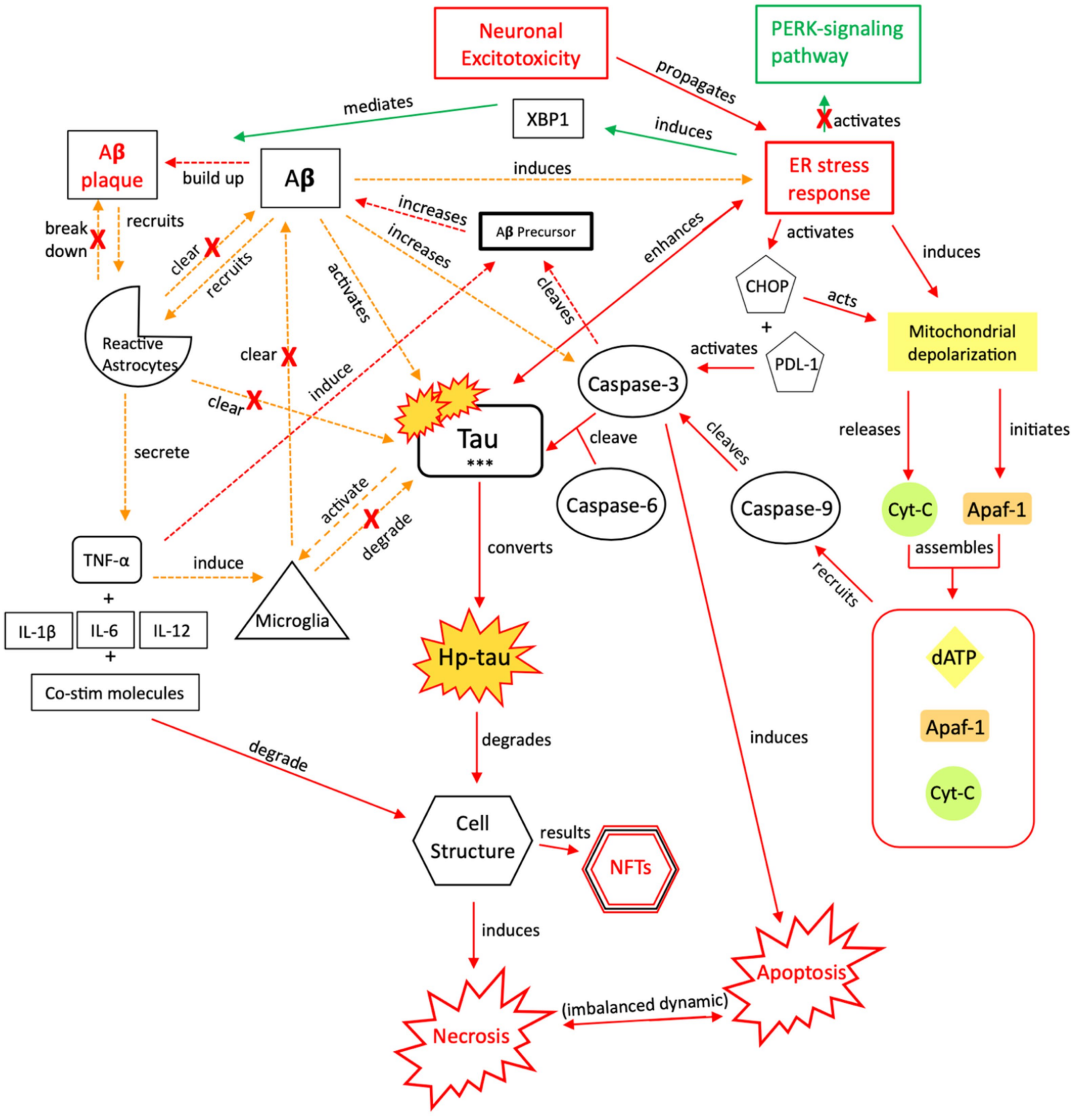
The injurious response of Aβ, aka its failed “last ditch effort” to downregulate propagation of toxic tau and rebalance apoptotic-necrotic signaling dynamics. Due to accumulated toxic tau aggregates and dysregulated tau clearance by reactive astrocytes and microglia, pro-death signaling mechanisms become favored over cellular preservation signaling. However, a recurrent, reactive ER stress response leads to an imbalance of apoptotic-necrotic signaling and enhances atypical tau phosphorylation. Further, Aβ precursor protein is cleaved by caspase-3, and Aβ concentrations increase, further propagating the ER stress response (88, 154, 155). Due to the Aβ precursor overexpression and increased Aβ production, defective mitochondria are produced, mitochondrial dynamics are altered, and their trafficking is reduced, leading to further intracellular Ca^2+^ influx and apoptotic-necrotic imbalance (166). However, the ER stress response also has neuroprotective effects, inducing selective transcription factor XBP1, which mediates Aβ plaque formation (88). Simultaneously, Aβ directly recruits reactive astrocytes and indirectly recruits microglia, through TNF-α and pro-inflammatory signaling, which cluster around Aβ plaques to clear them (157, 167, 168). Yet, the induction of pro-inflammatory signaling from astrocytic recruitment further induces Aβ precursor protein; increased Aβ concentrations result in increased atypical tau phosphorylation/hyper-phosphorylation and further ER stress response induction (169). Thus, reactive astrocytes have both neuroprotective and injurious effects (170). Continued apoptotic-necrotic signaling imbalance, degradation in cell structure, and NFT formation results from atypical activation of these pathways. PERK, protein kinase R-like ER kinase; Aβ, amyloid beta; XBP1, X-box binding protein 1; TNF, tumor necrosis factor; IL, interleukin; Co-stim, co-stimulatory (molecules); Cyt-c, cytochrome-c; Apaf-1, apoptotic peptidase activating factor-1; dATP, deoxyadenosine triphosphate; NFTs, neurofibrillary tangles; Red = Pro-death signaling, Green = Neuroprotective signaling, Orange = Aβ-involved signaling; o-tau, tau oligomers; t-tau, total tau; p-tau, phosphorylated tau; *** = O-tau, t-tau, p-tau. X = reduction/down-regulation. Solid line = signaling cascade induced/propagated by the ER stress response and tau; dashed line = signaling cascades resulting from Aβ involvement.

**Figure 9:**
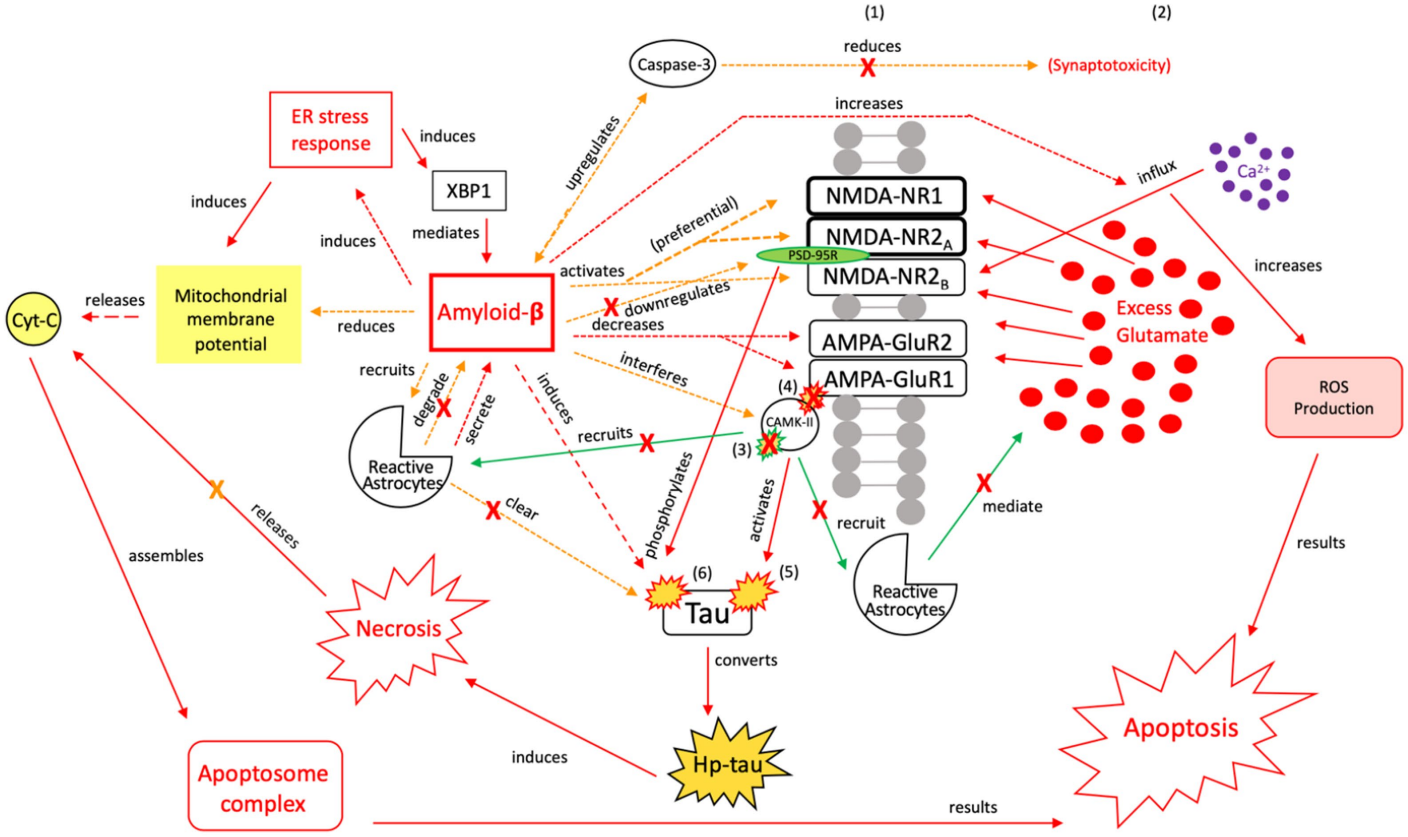
The injurious response of Aβ, aka the failed “last ditch effort” to revert the cell to pro-apoptotic signaling and rebalance apoptosis-necrosis, due to recurrent or sustained Aβ signaling. Unlike neuroprotective Aβ responses, preferential activation of NMDA-R1 and-2A/B receptor subunits by Aβ (171, 172), and their increased surface expression regulated by PSD-95, adversely affects channel assembly and conductance (173), promoting further neuroexcitotoxicity, atypical tau phosphorylation, and increased susceptibility to Aβ (164). Unsuccessful toxic tau aggregate and Aβ breakdown by microglia [seen in (A)] propagates the injurious effects of Aβ-associated tau seeding and propagation (133). In the presence of dysregulated tau and Aβ mechanisms, as well as dysregulated microglial and reactive astrocytic clearance, the failure to reduce neuroinflammation and excitotoxic propagation results in a transition from neuroprotection to neurodegeneration. We posit that this point marks the transition from a injurious mechanism to a more widespread neurodegenerative process. XBP1, X-box binding protein 1; Cyt-c, cytochrome-c; ROS, reactive oxygen species; Red = Pro-death signaling, Green = Neuroprotective signaling, Orange = Aβ-involved signaling; X = reduction/down-regulation. Solid line = signaling cascade induced/propagated by the ER stress response and tau; dashed line = signaling cascades resulting from Aβ involvement. (1) = Neuronal membrane, (2) = Synaptic cleft, (3) = CaMK-II-autophosphorylation, (4) = CaMK-II-Glutamate receptor phosphorylation, (5) = CaMK-II-tau-phosphorylation, (6) = PSD95-NMDA receptor complex-tau phosphorylation.

With chronic pathology, Aβ concentrations are also increased by caspase-3-mediated APP cleavage and an imbalanced ER stress response (88, 154, 155). We posit that, in response to recurrent or severe injury, sustained Aβ signaling is a “last ditch effort” by the cell to restore cell death signaling and reduce the injurious effects of an imbalanced ER stress response and atypical tau (Figures 6, 7). Although Aβ induction increases plaque formation, it also has neuroprotective effects, recruiting additional reactive astrocytes and microglia for toxic aggregate breakdown (157, 167, 168, 170). However, if the cell cannot degrade toxic tau and Aβ aggregates and restore cell death signaling, Aβ’s relationship with tau further transitions the NPT response to a neurodegenerative process because it prevents tau from appropriately binding to microtubules and induces atypical tau phosphorylation (154, 156, 171, 172) (Figures 8, 9).

In both typical functioning and in response to acute neuronal injury, we postulate that tau and Aβ signaling processes occur in parallel. In acute neuronal injury, however, we propose greater initial reliance on tau signaling in comparison to Aβ signaling, in avoidance of necrotic processes and reorientation toward cellular preservation and stabilization. With recurrent or severe neuronal injury, we postulate that the “last ditch effort” of Aβ indicates a “cellular switch” to greater reliance on Aβ signaling, for the purpose of activating apoptotic signaling and limiting neurotoxic spread. If the underlying injurious pathology is not reduced/halted, the result is a transition of the “at risk” neuroprotective response to one of neurodegeneration.

The ER stress response can induce apoptotic signaling cascades (169) in addition to promoting Aβ formation. Aβ formation comes with several costs, in that Aβ will activate pro-inflammatory responses and caspase-3 activity, in attempts to revert the cell to pro-apoptotic signaling; however, increased caspase-3-selective tau cleavage by Aβ and dysregulated mitochondrial production and recruitment results in further tau-related toxicity and an imbalanced intracellular dynamic (156, 157). While caspase-3 typically promotes tau cleavage and phosphorylation during apoptotic signaling, Aβ increases aberrant caspase-3 activity during necrosis (Figures 6, 7) (155). Hence, Aβ initiates atypical tau cleavage. It renders tau increasingly susceptible to hyperphosphorylation and toxic aggregates, because it atypically alters tau at specific phosphorylation sites (154), ultimately leading to microglial injury and neurotoxicity (Figure 8) (156). In AD, soluble Aβ induces tau hyperphosphorylation in hippocampal neurons, disrupting microtubule stability. De-phosphorylation of Aβ-induced p-tau results in the restoration of tau microtubule binding capacity (154), suggesting that the process is at least partially reversible, and suggests some initial benefit of Aβ formation.

Extracellular insoluble Aβ aggregates, however, are associated with neurotoxicity and degeneration (155). Both soluble and insoluble Aβ_1-40_ and Aβ_1-42_ levels are elevated in patients with AD compared to typical aging brains (174). The soluble forms comprise the greatest proportion of total Aβ in typical aging brains but the lowest in AD brains (174). Acute cell death is highly dependent upon the relationship between soluble Aβ and soluble cytoplasmic tau, which can propagate extracellularly (175). The relationship between Aβ and tau suggests that each can act on the other in a negative feedback loop, triggering the transition from non-toxic to toxic aggregates (175). Therefore, it is possible that soluble Aβ reflects typical brain functioning, but with neuronal injury, neurons are “at-risk” for soluble toxic tau formation and toxic tau/Aβ aggregate propagation extracellularly, resulting in an eventual transition to an insoluble state. This, in turn, reduces the proportion of soluble to insoluble Aβ and soluble phosphorylated to abnormally phosphorylated tau, further transitioning the mechanism to one of eventual degeneration (176).

Toxic Aβ accumulation results from several mechanisms, with prominent roles of microglia and astrocytes. Similar to tau, Aβ clearance requires microglial and reactive astrocytic degradation (Figure 6) (157). Aβ plaques can result from microglial dysregulation and increased Aβ-induced caspase-3 activity, as caspase-3 cleaves APP-β (177). Aβ also activates reactive astrocytes, which cluster around Aβ plaques (Figure 6) (167, 168). The astrocytes secrete interleukins and TNF-α, promoting further inflammation to break down Aβ (167, 168), however, these pro-inflammatory proteins also induce APP-β (178, 179), resulting in increased Aβ concentrations. Further neurodegeneration can also occur due to astrocytic secretion of Aβ (Figure 9) (180). Aβ deposits were found in the hippocampus with progression to the cortex prior to the formation of NFTs in a transgenic AD model, supporting neurodegenerative signaling cascades outlined in Figure 5 (181). Aβ deposits were also found in ~30% of severe TBI cases postmortem (182, 183). This, coupled with Aβ-promoted tau cleavage (9), indicates a relationship between amyloid-β, tau, and NFTs.

*In vitro* and *in vivo*, microglia clear soluble extracellular Aβ via micropinocytosis, in which successful uptake and degradation depends on actin and tubulin dynamics (184). Inflammatory processes promote signaling cascades and the recruitment of microglia to initiate soluble Aβ uptake and degradation (157). In an acute injury model, this process is postulated to be neuroprotective. However, with recurrent or sustained injury, this process may be dysregulated due to unstable actin/tubulin dynamics and imbalanced ATP involvement, leading to further neural injury. The transition from soluble to insoluble Aβ has yet to be fully understood. However, data suggest that a progressive Aβ transition from soluble to insoluble takes place in the ER/intermediate compartment pathway, and that the degree of insolubility correlates with overexpressed APP-β concentration (185). The uptake and degradation of insoluble Aβ, comprised of neurotoxic, soluble Aβ oligomers, occur through different endocytic mechanisms that are microglia and astrocytic receptor mediated (186–188). Further, simultaneous intra-astrocytic accumulation of soluble and neurotoxic Aβ for degradation promotes vesicle-induced neuronal apoptosis (189). Resulting from cell death, cellular contents, including neurotoxic Aβ, are released into cytoplasm and quickly re-phagocytosed by surrounding neurons. In acute injury, this process would be neuroprotective for the prevention of necrosis; however, with recurrent injury, it is a mechanism for further neurotoxic propagation and eventual systemic degradation.

Aβ activity disrupts cellular integrity, but we posit that Aβ attempts to minimize neurodegenerative damage by targeting NMDA/AMPA receptors and mitochondrial membrane potential (MMP) as part of a “last ditch effort” to reactivate apoptotic signaling (Figures 6, 7). Aβ recruits reactive astrocytes to compensate for microglial dysregulation and clear toxic Aβ (Figure 6). However, because shared biochemical mechanisms associated with neuronal homeostasis and cell death are dysregulated, and neuroprotective mechanisms such as reactive astrocytic phagocytosis of Aβ are functioning abnormally (180), these pathways promote further excitotoxic signaling and neurodegeneration (Figures 8, 9). If Aβ is not properly cleared, it can cause further atypical tau hyperphosphorylation, microtubule destabilization, and assembly of tau into filament structures seen in AD (190).

Aβ oligomers preferentially activate NMDA NR1/NR2A receptor subunits, which initiate LTP and regulate NMDA NR2B-mediated calcium influx (191). Aβ oligomers can induce a rapid increase in intracellular Ca^2+^ via NR2B influx and cause mitochondrial damage leading to hippocampal cell death (191). Aβ peptides interfere with CaMK-II activity and decrease AMPA receptor trafficking, leading to atypical synaptic distribution and LTP/LTD disruption (171, 172). The Aβ and NMDA relationships may explain a sustained excitotoxic response seen post-TBI/post-seizure. Due to sustained NR1/NR2A responses to high frequency stimulation, disrupted NR2B-mediated calcium influx, and diminished AMPA receptor activity (171, 172), downstream effects of RIR continue, along with a failure to clear excess synaptic glutamate (Figure 3). AMPA receptors are crucial for synaptic plasticity, learning, and memory (192, 193). Loss of AMPA receptors results in diminished synaptic transmission, long-term depression, and difficulties with learning and memory (193). In both brain tissue from AD patients and Aβ-treated neurons, there are significant decreases in AMPA receptor densities, with higher receptor turnover (194). In the presence of Aβ, decreased AMPA receptor expression and greater receptor turnover may be early indicators of atypical mechanistic changes associated with AD and resultant cognitive decline. If Aβ is acutely activated, we posit that the cell reorients to apoptotic signaling, minimizing injurious effects of Aβ; however, chronic Aβ activation further imbalances apoptotic-necrotic signaling and initiates a transition of this “last ditch effort” from injurious to neurodegenerative.

Several mechanisms act in concert to increase phosphorylation of tau in the setting of repeated injuries. (1) Aβ induces caspase-3 activation (195) (Figure 5). (2) Aβ-42 reduces MMP in cortical neurons (122, 146, 196), thereby increasing ATP production and cyt-c release. Cyt-c mediates caspase-3 activation that leads to tau cleavage and phosphorylation (197). (3) Endogenous tau interacts with the PSD95-NMDA receptor complex, which selectively phosphorylates tau (198). To efficiently kill the cell via apoptosis, Aβ must activate alternative apoptotic pathways while reducing the tau response. (1) Caspase-8 recruitment by Aβ mediates the relationship between Aβ and caspase-3, resulting in decreased synaptic excitotoxicity and a reorientation toward apoptotic signaling (163). (2) NMDA NR1/NR2A receptor activity affects downstream ROS production resulting in apoptosis (163, 198). (3) Aβ downregulates the PSD95-NMDA receptor complex, decreasing tau phosphorylation. In cultured cells, Aβ-induced apoptosis increased reactive oxygen species (ROS) production but not hp-tau (165). While ROS-produced apoptosis has detrimental effects, as a “last ditch” neuroprotective effort of Aβ, it limits further hp-tau and NFT formation. Limiting the effects of tau and Aβ toxicity is predicated on the acute nature of this response and treatment of the underlying pathology to avoid long-term neurodegeneration.

### 4.1 Summary of NPT, NIT, and Aβ hypotheses

Tau phosphorylation antagonizes apoptotic processes in response to increased ER stress and imbalanced homeostatic dynamics (147). Tau hyperphosphorylation is a reactive response activated when faced with apoptotic cell death (120, 129). The build-up of hp-tau, therefore, could represent a failed neuroprotective mechanism. NFTs, a hallmark of tauopathies, form as a downstream result of the RIR and NPT pathways. In addition to containing hp-tau, NFTs contain active caspase-6, caspase-6-cleaved tau, and Aβ, further supporting that NFTs are the end result of a neuronal degradation pathway – one that initially includes a neuroprotective pathway preferred by the cell over acute apoptotic death (9), but over time, becomes overwhelmed by the accumulation of repeated injuries.

The NPT response suggests that excess tau is phosphorylated in attempts to preserve cellular integrity in the short-term. In response, microglia and reactive astrocytes are triggered to reinstate homeostasis and break down intracellular tau into non-toxic isoforms. However, in the setting of repeated injury, when excess tau phosphorylation exceeds microglial and astrocytic capacity for tau degradation, toxic tau accumulates. This, along with aberrant tau cleavage, aggregation, and hyper-phosphorylation, propagates a dysregulated microglial response. To combat this, the toxic tau must be expelled from the cell and is done so through exosomal packaging and secretion.

We posit that the response mechanism is neuroprotective to the point of halting apoptosis, phosphorylating tau, and clearing tau via microglia and reactive astrocytes, and that it would continue to be neuroprotective if repetitive seizures or head injuries did not (1) lead to Aβ accumulation and its production of toxic tau and (2) outpace the ability to clear tau. Because epilepsy and repeated TBIs are plagued with recurrent cellular injury and ER response activation, however, a buildup of cleaved, phosphorylated, and hyperphosphorylated tau results in toxic tau aggregates. These toxic aggregates are then propagated to surrounding neurons, adversely affecting these neighboring neurons and increasing the likelihood for localized neuronal degeneration. The extracellular leakage of toxic tau also contributes to NFT formation and induces tauopathy-related necrosis, transitioning the mechanism over time from neuroprotective to neurodegenerative.

The role of Aβ is pivotal in the development of neurodegeneration. Aβ induces tau phosphorylation, contributing to toxic tau aggregates that cannot be cleared by microglia and reactive astrocytes. Additionally, microglia cannot clear the excess Aβ, leading to inflammatory signaling, excitotoxicity, and Aβ plaque accumulation. Microglial and reactive astrocytic dysregulation results in further tau and Aβ leakage that contributes to injury. We posit that, although increased Aβ concentration has a deleterious effect on cellular integrity and microglial functioning, increased Aβ also reactivates preferential apoptotic signaling by targeting NMDA/AMPA receptor functioning, CaMK-II phosphorylation, astrocytic recruitment, and mitochondrial membrane permeability (Figure 6). Because glutamate transmission, apoptosis, and necrotic signaling share related pathways, this Aβ compensatory mechanism cannot differentiate between typical and atypical activation, such that excitotoxicity continues. Recurrent or sustained activation of these mechanisms results in the necrotic cell death seen in tauopathies.

## 5 Clinical correlations: tau and TBI

Early studies lacked an association between TBI and cerebrospinal fluid (CSF) p-tau levels, likely because of insufficient sensitivity of the assay, requiring development of novel techniques (92, 199). An enhanced immunoassay using multi-arrayed fiber optics (EIMAF) detected acutely increased t-tau and p-tau levels in brain and blood following CCI in rodents and in CSF following severe TBI in humans. T-tau and p-tau levels remained significantly elevated during the chronic stage of CCI in rodents. While t-tau and p-tau levels decreased during the chronic stage of severe TBI in humans, elevated levels were still detected in subsequent months post-injury. T-tau levels approached normal limits approximately one-month post-injury, while p-tau levels remained elevated six months post-injury (200). EIMAF also demonstrated increased p-tau levels, t-tau levels, and p-tau/t-tau ratios in individuals with acute or chronic TBI compared to healthy controls (201). Using a single-molecule enzyme-linked immunosorbent assay (SIMOA), blood t-tau levels were greater in professional hockey players across multiple time points post-head injury (from one to 48 h) compared to preseason (pre-injury) (91). Recent studies have also measured tau within exosomes isolated from plasma (202, 203). This technique has been applied in remote repetitive TBI, with elevated exosomal t-tau and p-tau levels negatively correlating with neuropsychological measures (202, 203).

Tau levels correlate with clinical recovery, with a negative association between CSF tau and clinical improvement (204). Ventricular CSF t-tau concentrations in the setting of severe TBI negatively correlated with clinical improvement over one year (205). Plasma p-and t-tau levels measured in patients ~24-h post-acute head injury were associated with short-and long-term outcomes; p-tau and p-tau/t-tau ratios in blood negatively correlated with recovery in participants with chronic TBI (201). Human data concur with a rat model, in which serum and CSF tau levels positively correlated with traumatic spinal cord injury severity and negatively correlated with locomotor function (206). These results support p-tau as a biomarker that reflects a broad picture of axonal injury, TBI severity, cognitive functioning, and long-term outcomes.

## 6 Clinical correlations: tau and AD

Pathological p-tau aggregation is a biomarker of neurodegeneration in AD. In a transgenic mouse model of AD, microglial activation occurs in a progressive fashion, correlating with increased tau hyper-phosphorylation and Aβ plaque accumulation (207). Human and animal models of AD and other dementias identify atypical tau processes that contribute to increased hyper-phosphorylation, microglial activation, NFT formation, and neurodegeneration (208), including genetic mutations and post-translational modifications (209–214). Atypical tau phosphorylation and APP mutations correlate with NFT formation in animal models and human AD (215, 216). In human AD brain tissue, tau pathology was divided into early and late stages, with tau deposition first observed in entorhinal cortex and hippocampus. Later tau aggregates correlated with cognitive decline (217). In human lateral temporal cortex obtained from late-stage AD brains, increased markers of the ER stress response correlated with decreased post-synaptic PSD-95 markers and increased tau (218).

Increased CSF t-tau levels were also found in patients with AD (219). Elevated CSF tau levels demonstrated a strong association with AD and improved discrimination of AD from other dementias, while Aβ levels failed to improve diagnostic accuracy (220). CSF p-tau181, 217, and 231 concentrations accurately predicted cognitive impairment in patients with AD, but not in patients with other dementias or controls (221). P-tau231 was the earliest detector of increased Aβ in AD pathology, preceding Aβ identification by position emission tomography (PET) (221). Further, increased levels of tau and decreased levels of Aβ_1-42_ in CSF were reported (222–226), highlighting their contrasting CSF profiles as biomarkers for AD. In plasma, tau levels were significantly higher in patients with AD compared to MCI patients and controls, however, use of plasma tau as a diagnostic test is not yet validated (227).

## 7 Clinical correlations: tau and epilepsy

A link between AD and temporal lobe epilepsy (TLE) is demonstrated by a bidirectional increase in risk, hippocampal damage (228), and cognitive deficits in both disorders, in part due to shared cortical networks, tau deposition, and amyloid pathology. Current research explores the influence of seizure activity on tau levels in brain, CSF, and blood, proposing that epilepsy is a tauopathy like AD and CTE – with proposed mechanisms of tau deposition including production during ictal and interictal activity, axonal sprouting and formation of aberrant connections in response to injury, cell death, physical injury during seizures, and decreased clearance (94). Studying the relationship between tau and epilepsy may address how seizure activity results in neuronal injury.

Limited data are available regarding tau levels in people with epilepsy. Hp-tau deposits were identified in resected temporal lobe tissue from patients with hippocampal sclerosis, evident in nearly 94% of cases and correlating with post-operative declines in verbal memory and naming, though this finding was not seen in all resection studies (94). In late-onset epilepsy of unknown origin, CSF t-tau levels were increased in comparison to controls, with t-tau and p-tau levels predicting onset of dementia (229). Elevated CSF t-tau and p-tau levels were detected in patients with status epilepticus when tested at a median of 72 h from admission (95). In the setting of status, t-tau levels positively correlated with medication resistance, status duration, disability, and development of chronic epilepsy (95). While a transient increase of CSF t-tau was reported within four days of a single, new-onset generalized convulsion, tau elevations in isolated or repeated seizures that respond promptly to medications are controversial (96, 97). Increased CSF t-tau levels were seen with symptomatic convulsions (of acute or remote etiology), but not in subjects with seizures of idiopathic or cryptogenic cause when levels were obtained within 48 h (98). CSF t-tau levels were decreased, and p-tau unchanged, when CSF was collected at least seven days after the last seizure, but seizure frequency was unknown (230). Blood–brain barrier disruption during seizures may release tau to the periphery, suggested by small, transient elevations of serum T-tau following convulsions (231). Studies of peripheral p-tau and exosomal analyses have not yet been applied to people with epilepsy, and the impact of epilepsy-related factors (e.g., seizure type, epilepsy duration) on peripheral tau levels is unknown.

The relationship between epilepsy and p-tau levels should be explored as a potential marker of neural injury severity and predictor of cognitive function and seizure control. Given the above similarities in injury pathophysiology between AD, TBI, and epilepsy, AD and TBI may serve as guides to identifying overlapping markers of neuronal damage and cognition.

## 8 Treating AD, TBI, and epilepsy: pharmacological interventions

### 8.1 Cytokine targets

A better understanding of tau deposition lends insight into AD, TBI, and epilepsy pathophysiology and presents possible targets for intervention. Potential approaches include neuroprotection, inhibition of inflammatory processes, and disruption of excitotoxic mechanisms. Trials focused on various portions of these pathways. In animal models of TBI, minocycline and statins demonstrate beneficial anti-inflammatory and neuroprotective properties, limit the expression of pro-inflammatory cytokines, and render cell death-associated astrocytes and microglia inactive (232–234). *In vitro* and *in vivo* rat brain TBI and immune system studies identified human-cultured mesenchymal stem cells coupled with purified immune cells as a promising treatment that increases production of anti-inflammatory ILs, while decreasing TNF-α (235–237). IL-34 selectively enhances microglial neuroprotective effects, homeostasis, and neuronal survival by promoting Aβ oligomeric clearance and inducing microglial enzymatic activity. These effects reduce oxidative stress without promoting neurotoxicity (61). Promotion of IL-34 receptor binding or activity may benefit those with recurrent seizures/TBI by enhancing microglial function.

### 8.2 NMDA receptor antagonists

Data regarding NMDA antagonists are mixed. Drugs like amantadine, a weak NMDA antagonist, are commonly used in acute brain injury rehabilitation, although supporting data are limited (238). In some TBI studies, NMDA receptor antagonists lacked efficacy and raised safety concerns (239). A trial of the competitive NMDA antagonist D-CCP-ene for the treatment of intractable focal-onset seizures led to severe adverse events in all eight subjects, including sedation, ataxia, depression, amnesia, and poor concentration (240). Seizure frequency worsened in three subjects and remained unchanged in four subjects; one participant demonstrated improved seizure frequency, yet experienced status epilepticus upon D-CCP-ene withdrawal (240). All subjects withdrew from participation, leading to premature termination of the study. However, in a large, randomized, double-blind, placebo-controlled trial of traxoprodil, an NMDA NR2B subunit antagonist, was found to be well-tolerated in adults with severe TBI; they demonstrated improved Glasgow Coma Scale outcomes 6-months post-injury compared to placebo (241).

In an animal model of hippocampal seizures, MK-801 decreased seizure severity at low doses (242). In 68 patients with super-refractory status epilepticus, ketamine infusions administered for a length of one to four days reduced seizure burden by 50% (243). Upregulated NMDA receptor trafficking in the post-synaptic membrane contributes to super-refractory status epilepticus; NMDA receptor antagonists like MK-801 and ketamine may be effective due to improved penetration of the blood brain barrier and maintain their function even in the presence of increased concentrations of intra-and extra-cellular glutamate (244–246).

Memantine, a low-affinity voltage-dependent uncompetitive NMDA antagonist, approved for use in AD, reduced tau phosphorylation and improved functional outcomes after repetitive mild TBI in adult mice (247). In patients with TLE, memantine improved cognition compared to donepezil (248). In a double-blinded, placebo-controlled trial, once-daily memantine significantly improved episodic memory and quality of life in patients with epilepsy, although confounded by reduced seizure frequency (248, 249). In contrast, in subjects with focal-onset seizures of unchanged frequency, memantine yielded no significant improvement in cognition compared to placebo (250). However, in an open-label extension phase, there were improvements in verbal memory, memory-related quality of life, and executive functioning (250). Overall, NMDA antagonists deserve further study in TBI, AD, and epilepsy (238, 241).

### 8.3 AMPA receptor antagonists

Alternatively, perampanel is highly selective for AMPA receptors and inhibits AMPA-induced calcium influx in rat cortical neurons (251). Pharmacological dampening of AMPA receptor function eliminated interictal-like activity in human lateral amygdala *in vivo*, without reducing AMPA receptor densities observed *in vitro* (252). It is efficacious for treatment of focal-onset seizures with a neutral cognitive profile in adult, geriatric, and pediatric patients (253–255). In a rat CCI model, perampanel preserved neurological function, inhibited apoptosis and microglial activation, reduced brain edema, and preserved blood–brain-barrier functioning post-injury, thereby protecting neuro-vasculature (256). It also reduced brain contusion volume and decreased expression of pro-inflammatory TNF-α and IL-1β (257).

The effects of perampanel on neurological functioning, inflammatory markers, and cognition in patients with AD has yet to be comprehensively studied, outside of isolated case reports. In a case study of an 89 year old woman with severe AD, intractable myoclonic epilepsy, and psychiatric symptoms of circadian rhythm disorder and irritability, perampanel improved both myoclonus and psychiatric symptoms (258). An additional case report demonstrated improved cognitive functioning in a patient with non-convulsive seizures and AD, supporting the case for early administration (259). In transgenic AD mice, inhibition of AMPA receptors by perampanel reduced hippocampal Aβ_40_ and Aβ_42_ levels and decreased levels of the soluble peptide APPβ by suppressing β-cleavage of APP (260). Further research is needed into the potential effect of perampanel in targeting Aβ pathology by reducing Aβ production in AD.

### 8.4 Metabotropic glutamate receptor treatments

Metabotropic glutamate receptors (mGluR) may also be a target of interest in generalized and focal seizures, as the group II and III mGluR agonists may decrease NMDA receptor function and the risk of excitotoxicity. Animal studies showed anticonvulsant effects of the group II mGluR agonist, DCG-IV, in models of limbic and generalized motor seizures (261–263). Anticonvulsant effects were also noted in DBA/2 rodent models using agonists that target group III mGluR_8_ and mGluR_4_, 4A (L-AP4, RS-4-PPG, and ACPT-1) and the antagonist, MPPG. These agents were not found to affect group I, which contribute to epileptogenesis (264, 265). However, mixed responses to mGluR-based treatments have been noted, with proconvulsant effects of group III agonists (L-AP4 and L-SOP) and the MGluR antagonist, MAP4 (266, 267). Further research is needed into safe and effective therapeutic concentrations of mGluR-targeting agents, as well as their role in seizure activity (266–270).

In our proposed RIR model, upregulation of selected NMDA receptors and downregulation of selected AMPA receptors occurs as a result of neuroinflammation in response to sustained or recurrent injury. As a result, there is an increased likelihood of seizure occurrence and atypically high concentrations of intra-and extra-cellular glutamate. At low doses, NMDA receptor antagonists can reduce seizure severity and frequency, but with mixed results. Higher doses, however, risk significant adverse effects. AMPA receptor antagonists, such as perampanel, may show greater promise due to their potential effects on hyperexcitability, underlying pathophysiology of neurodegenerative disorders, and tolerability. MGluR agonists and antagonists showed varied pro-vs. anti-convulsant effects with limited research into safe and effective therapeutic concentrations. Caution in targeting glutamate receptors is warranted.

### 8.5 Monoclonal antibody treatments

Anti-amyloid monoclonal antibodies, such as lecanemab and aducanumab, represent another treatment approach, possibly as maintenance drugs to slow the progression of cognitive decline over the course of the disease. Lecanemab demonstrated high affinity binding to soluble Aβ, and particularly to Aβ soluble protofibrils, which are seen in early AD (271–273). Approved for use in Alzheimer’s disease (274), lecanemab reduced Aβ markers and moderately slowed cognitive decline over 18 months compared to placebo (271, 272), although its effectiveness has been questioned. In a transgenic mouse model, aducanumab decreased both soluble and insoluble Aβ in a dose-dependent manner (275). To evaluate the safety and efficacy of aducanumab in reducing cognitive decline in patients with MCI and mild AD, two large, double-blind, placebo-controlled studies were conducted. Results indicated that aducanumab was associated with dose-dependent amyloid related imaging abnormalities (ARIA), with cerebral edema and increased risk of intracerebral hemorrhage, particularly in ApoE-ɛ4 carriers (276). Infusion-related reactions and other adverse events (including ARIA) make anti-amyloid antibodies a controversial approach in the setting of uncertain benefits. Lecanemab and aducanumab have not yet been tested in patients with TBI or epilepsy, and safety and efficacy clinical trials for both drugs are on-going.

### 8.6 Tau-centric treatments

Reduction of tau levels showed promise in tau-expressing transgenic mice with repetitive mild CHI. Mice were treated with kinase-targeting lithium chloride and R-roscovitine, leading to p-tau reduction that correlated with improved cognition (200, 277). Alternatively, phosphatases dephosphorylate toxic tau into non-toxic isoforms. Phosphatase 2A (PP2A) dephosphorylates hp-tau, but PP2A activity is decreased in AD brain (278, 279). In AD, GSK-3 activation inhibits PP2A (280), and PP2A inhibitory proteins (inhibitor-1 and -2) are upregulated (281). Pharmacological interventions that inhibit GSK-3, such as SAR502250 (282), or support mRNA-based downregulation of PP2A inhibitors-1/2, are promising approaches (281). Drugs for approved for other indications may also be “repurposed” given their effects on tau. Suvorexant, an FDA-approved drug for insomnia, reduces tau phosphorylation at selective sites such as −181 and decreases Aβ concentrations compared to placebo (283); its use should be investigated in other disorders. Angiotensin receptor blockers, FDA-approved for hypertension, have anticonvulsant effects in rats (284–286) and decrease incidence of epilepsy in humans (287), while decreasing CSF t-tau and p-tau in MCI patients (288) and improving cognition in hypertensive older adults with early executive impairment (289) and prodromal AD (290). These results support the need to further investigate the safety and efficacy of tau-targeting drugs in epilepsy.

### 8.7 ER stress response inhibition

Based on our proposed mechanism, drugs that impair the PERK pathway would have injurious effects. In a mouse TBI model, for example, inhibition of the PERK signaling pathway by GSK2606414 exacerbated immature cell loss, dendritic loss, and cell death (26).

Conversely, pharmacological upregulation of the PERK pathway may be an effective treatment target to avoid atypical ER stress response activation, reduce tau phosphorylation by ER stress response signaling (75), and mediate tau hyper-phosphorylation and Aβ neurotoxicity (291).

Drugs that target the ER stress response cell death pathways may also aid in the restoration of intracellular homeostasis, apoptotic-necrotic signaling dynamics, and ER folding capacity. In a rat lateral fluid percussion model of TBI, administration of the ER stress response inhibitor, salubrinal, 30 min prior to injury significantly reduced the ER stress response, promoted mitochondrial functioning, and inhibited downstream apoptotic signaling (292). In a mouse model of autosomal dominant lateral TLE, 4-phenylbutyric acid restored LGI1 protein function and reduced seizure susceptibility (293). In a mouse model of epilepsy, taurursodiol also reduced seizure susceptibility and mitigated repeated stress-induced neurodegeneration (294). In reducing seizure susceptibility, the likelihood of repeated or chronic activation of the ER stress response and tau-induced pathways decreases. This benefits the cell by favoring restoration of homeostasis, PERK pathway activation, and tau-involvement for maintenance of cellular dynamics; this also reduces the likelihood of repeated/chronic activation of Aβ, resulting in avoidance of irreversible or long-term neurodegeneration.

The numerous proteins and pathways involved in the brain’s inflammatory response make it challenging to identify the most appropriate target. Development of inflammatory modulators must also consider that acute inflammation can serve to protect neuronal integrity and avoid cell death, while chronic inflammation may decrease the likelihood of maximal recovery and cell survival. Further research is needed to find preventative and therapeutic agents for AD, TBI, and epilepsy.

## 9 Conclusion

AD, TBI, and epilepsy disrupt neuronal function and promote atypical response signaling. This review examined inflammatory and excitotoxic pathways common to AD, TBI, and epilepsy, the role of the ER stress response in the face of excitotoxicity, and tau and Aβ signaling. We proposed a mechanism by which these pathways can lead to tau deposition. We posit that tau accumulation represents an attempt to shunt the injury response from apoptosis toward neuroprotective signaling that preserves the cell, in attempts to restore homeostasis. This could be viewed as an acute “neuroprotective” response, although, if the underlying pathology is not treated, its recurrent or sustained activation will result in neurodegeneration. Our proposed mechanism supports the case for early intervention. In patients with AD, we must identify risk factors that impact tau and Aβ processes prior to the appearance of cognitive decline. In patients with TBI, this means reducing the likelihood of recurrent injury, reducing injury severity through preventative measures, and providing ample recovery time. In patients with epilepsy, we need to identify the underlying etiologies and reduce seizure frequency and severity. These pathways may present targets for intervention in AD, TBI, and epilepsy. Studies that examine mediators of these signaling cascades are needed.

## Data availability statement

The original contributions presented in the study are included in the article/supplementary material, further inquiries can be directed to the corresponding author.

## Author contributions

SM: Conceptualization, Investigation, Methodology, Visualization, Writing – original draft, Writing – review & editing. BAL-M: Conceptualization, Funding acquisition, Methodology, Supervision, Visualization, Writing – review & editing.
